# Papillomavirus Infectious Pathways: A Comparison of Systems

**DOI:** 10.3390/v7082823

**Published:** 2015-08-04

**Authors:** Jennifer Biryukov, Craig Meyers

**Affiliations:** Department of Microbiology and Immunology, The Pennsylvania State University, 500 University Drive, Hershey, PA 17033, USA; E-Mail: jhopkins@hmc.psu.edu

**Keywords:** human papillomavirus (HPV), virus, native virions (NV), virus-like particles (VLPs), pseudovirions (PsV), quasivirions (QV)

## Abstract

The HPV viral lifecycle is tightly linked to the host cell differentiation, causing difficulty in growing virions in culture. A system that bypasses the need for differentiating epithelium has allowed for generation of recombinant particles, such as virus-like particles (VLPs), pseudovirions (PsV), and quasivirions (QV). Much of the research looking at the HPV life cycle, infectivity, and structure has been generated utilizing recombinant particles. While recombinant particles have proven to be invaluable, allowing for a rapid progression of the HPV field, there are some significant differences between recombinant particles and native virions and very few comparative studies using native virions to confirm results are done. This review serves to address the conflicting data in the HPV field regarding native virions and recombinant particles.

## 1. Introduction

Human papillomaviruses are the etiologic agent of cervical cancer and other anogenital and oral cancers [1,2,3,4]. To date, over 150 types have been identified. Cervical cancer is the third most common cancer in women worldwide, causing a quarter of a million deaths per year [5]. All HPV types replicate within epithelium, however, they are subdivided based on their ability to infect either mucosal or cutaneous keratinocytes. HPVs that infect mucosal keratinocytes are further sub-divided into low-risk and high-risk types. Low-risk HPVs cause benign lesions such as condylomas or warts while high-risk types cause malignant neoplasms such as cervical cancer [6,7]. The most significant risk of developing cervical cancer is infection with a high-risk type. High-risk types such as HPV16, HPV18, HPV31, HPV45, and HPV58 have a strong link with malignant progression [6,7,8].

HPV virions consist of an icosahedral capsid containing histone-associated dsDNA. The viral genome is circular and approximately 8000 bp in length. There is an average of 8 open reading frames (ORFs), which are divided into early and late gene expression classes. HPV replication is tightly linked to the differentiation program of host epithelial cells. Infection occurs in basal epithelial cells via microabrasions. Once infected, early non-structural proteins involved in activities such as genome maintenance and transcription activation are expressed. As the daughter cells divide and become increasingly differentiated in the suprabasal layers, viral genomes are amplified, the late structural proteins are expressed, and new virions assembled [9,10,11,12,13].

Because the HPV life cycle is tightly linked to the host cell differentiation, the virus has been hard to grow in culture. Therefore, systems to create viral particles bypassing epithelial differentiation were developed. Recent research studying the HPV life cycle including infectivity, transmission, and viral structure predominantly relies on the use of recombinant papillomavirus particles. These recombinant particles such as virus-like particles (VLPs), pseudovirions (PsVs), and quasivirions (QVs) all bypass the need for stratifying and differentiating human epithelium. While data collected using recombinant particles has allowed for a rapid progression of the field of HPV research, there are few comparative studies utilizing native virions produced in organotypic raft culture, xenografts, or native tissue to confirm results in the context of stratifying and differentiating human epithelial tissue.

**Table 1:** A comparison of the advantages and disadvantages of the different types of papillomavirus particles.

HPV Particle Type	Method of Production	Particle and System Advantages	Particle and System Disadvantages
Native Virions	Organotypic raft culture system	Contains the full HPV capsid as well as full HPV genome Assembles and matures over a period of 10–20 days in tissue	Expensive Slow production time
Virus Like Particles (VLPs)	Transfection based system	Quick and inexpensive particle production	Codon modification of L1 and L2 is necessary for production Over-expression system Particles produced in non-relevant cell lines Particle assembly within 48 hours Maturation occurs overnight Deletion of in frame methionines upstream of the consensus methionine in L1 VLPs contain no genomes, which may alter particle structure
Pseudovirions (PsVs)	Transfection based system	Quick and inexpensive particle production Ability to track cellular infectivity	
Quasivirions (QVs)	Transfection based system	Quick and inexpensive particle production Contains L1, L2, and a full HPV genome Closest to NVs—retains majority of cell surface exposed conformational epitopes	

The main focus of this review will be to compare native virions produced in stratifying epithelium to recombinant particles (Table 1). All aspects of the viral life cycle including particle synthesis, maturation, attachment/entry, and infectivity will be evaluated. While much of the data generated utilizing recombinant particles has served to advance the field, there are many instances of conflicting results when compared to data generated utilizing native virions. A fraction of the conflicting data is likely due to utilization of different cell lines, particle maturation, and the multiplicity of infection used. Additionally, much of the research has been done using recombinant HPV16 with the assumption that what holds true for one HPV type is true for all HPV types. However, some recent data suggests that this may not be the case [14,15]. We hope to highlight not only the necessity for confirmatory studies but also the importance of studying more than one HPV type.

## 2. Papillomavirus Particle Production

**Figure 1:**
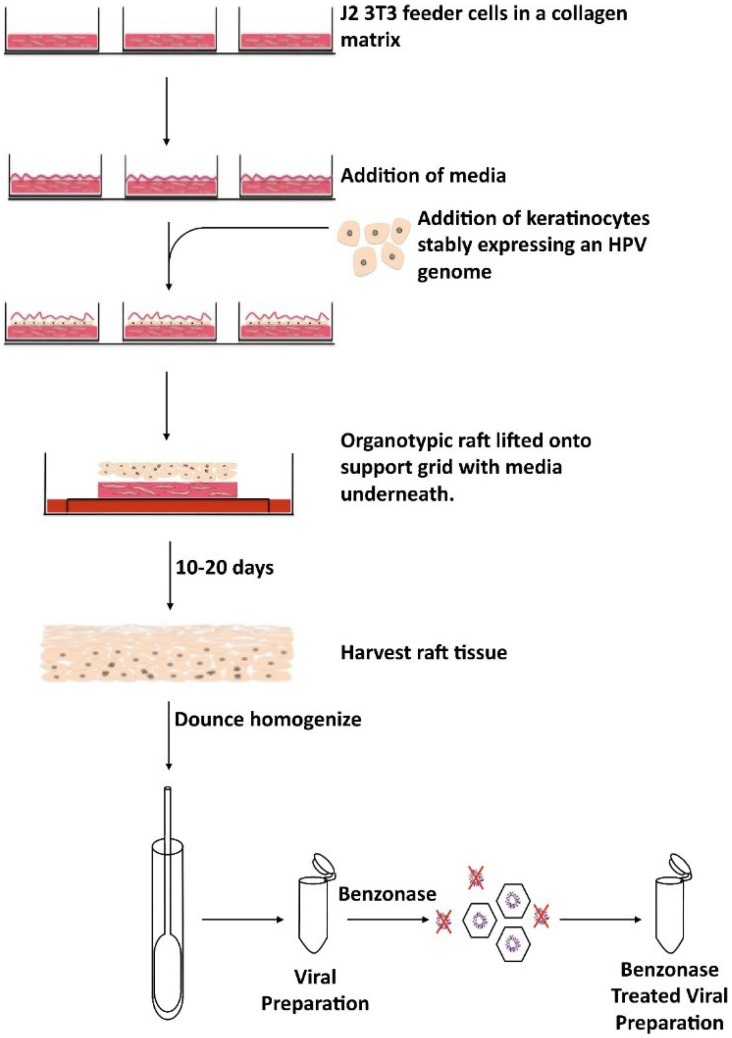
Schematic of native HPV virion production. HPV-positive cells are seeded on top of collagen plugs comprised of collagen and J2 3T3 feeder cells. Once the cells are have grown to confluence, the plugs are lifted onto a support grid. The rafts are then fed via diffusion from media underneath the grid. After 10–20 days, the tissue is harvested, homogenized, and benzonase treated to get a final viral preparation.

Organotypic raft culture is the only system outside of the xenograft system that allows for the production of infectious HPV in its natural environment, a differentiating epithelium [9,16,17,18]. This *in vitro* system preserves the molecular events that occur during differentiation with the convenience of not having to use animals such as in the xenograft system. In the organotypic system, human keratinocytes that stably maintain HPV viral genomes are grown above a dermal equivalent made of fibroblast cells embedded in a collagen matrix. Keratinocytes grow at the air liquid interface and are fed via diffusion from cell culture media below the dermal equivalent [9]. After a period of 10–20 days, tissue is harvested and homogenized to generate a virus stock (Figure 1). This system allows for the study of genetic mutants in the natural life cycle as genomes can be mutated, electroporated into keratinocytes, and grown in raft culture for the production of mutant virions. One drawback to using this system is the increased cost for virion production. Additionally, instead of producing particles in a matter of days, the raft culture system takes 3–4 weeks. Overall, this system allows researchers to examine, temporally and spatially, all parts of the viral life cycle including viral genome amplification, late gene expression, virion assembly, maturation, and infectivity.

**Figure 2:**
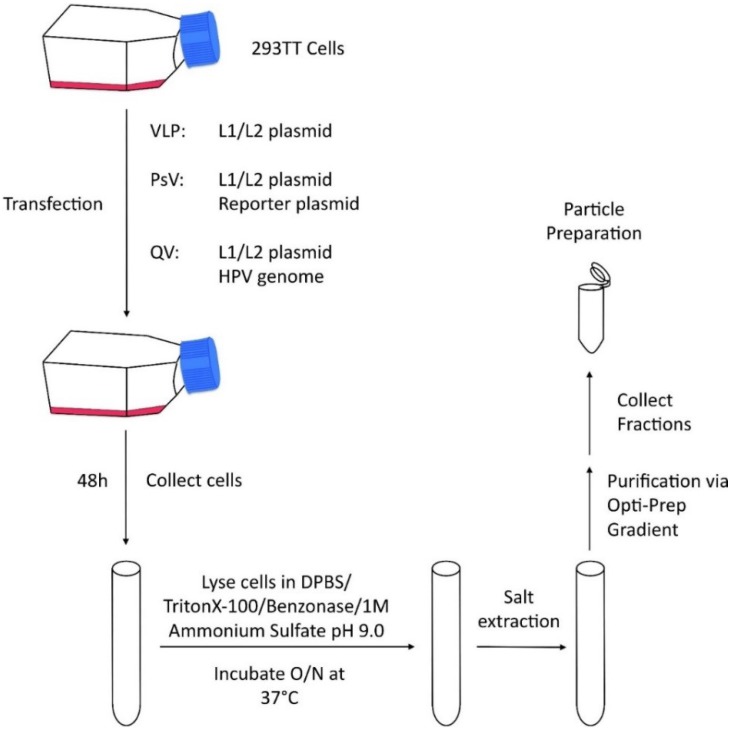
Schematic of recombinant HPV particle production. 293TT cells are transfected with a L1/L2 plasmid (VLPs), a L1/L2 plasmid and a reporter plasmid (PsVs), or a L1/L2 plasmid and a full HPV genome (QVs). Forty-eight hours post transfection, cells are harvested, lysed, and incubated at 37 °C overnight in the presence of cell lysate. Particles are then purified on a gradient.

The increased cost and technical challenges associated with acquiring virions from either an *in vivo* or *in vitro* system created a need for the development of the recombinant system (Figure 2). The basis of recombinant particle production comes from the fact that expression of either L1 alone or L1 and L2 together results in the self-assembly of proteins into viral capsids called VLPs [19,20,21,22,23,24,25]. For production of PsVs, in addition to the capsid proteins, a reporter plasmid encoding GFP or other reporter is co-transfected to function as a mock genome. These particles allow for researchers to easily track cellular infectivity [26,27]. Finally, when the full HPV genome is transfected into cells along with expression vectors for the capsid proteins, the particles are called QVs [26,27]. VLPs, PsVs, and QVs are assembled over a period of 48 h in monolayer culture and do not go through the natural maturation process. QVs represent the closest resembling recombinant particle to native virions, resembling NVs when compared by cryoelectron microscopy. The QV particles also retain the majority of surface exposed conformational dependent epitopes [28,29,30,31,32]. It is unclear if there are any structural differences between recombinant particles and NVs that might affect the biology of the virus. Due to the ease and low cost of production, recombinant particles remain the main tool used to study HPV structure, assembly, entry, and infectivity.

While the use of recombinant particles allows for rapid collection of data, there are some caveats to this system of particle production that should be considered. The first being that expression plasmids utilized to synthesize L1 and L2 have been codon optimized to remove rare codons and negate any possible limitations in protein expression [33,34]. While this does not change the protein sequence, this can affect the speed at which proteins are translated, leading to an increased chance of errors in the final amino acid sequence, or could affect protein folding [35]. Additionally, expression of proteins in non-keratinocytes, and especially in prokaryotic cells, could lead to a change in the post translational modifications in the protein. When comparing HPV6b L1 produced using a recombinant baculovirus in Sf9 insect cells or using a recombinant vaccinia virus in kidney cells, differing post-translation modifications were observed. Specifically, L1 produced in the Sf9 cells presented with several post-translationally modified variants, both threonine and serine residues were phosphorylated compared to just serine residues in the L1 produced in the kidney cells, and Sf9 produced L1 incorporated with both mannose and galactose whereas kidney cell produced L1 incorporated with only galactose [36]. While there are likely differences, studies to look at the differences in post-translational modifications between raft-culture derived virions and recombinant particles have not been done and the possibility of these differences should be kept in mind when drawing conclusions from experiments.

In the recombinant particle system, the L1 in all recombinant particles begins at a consensus methionine that was found when aligning the L1 N-terminal open reading frame (ORF) of many papillomaviruses. However, some HPV types, such as HPV16 and HPV18 actually have upstream, in frame, methionines that allow for the production of more than one L1 species. Specifically, HPV16 has one upstream methionine that allows for production of an L1 26 amino acids longer then L1 started at the consensus methionine and HPV18 has two upstream methionines enabling production of L1 proteins that are 61 and 26 amino acids longer then the L1 produced from the consensus methionine. These methionines were removed from the expression plasmids utilized in the recombinant system because only production of L1 from the consensus methionine allowed for efficient expression of L1 and production of particles [37]. A HPV18 NV 35 amino acid deletion mutant, deleting the region from the upstream methionine to the consensus methionine, was produced and tested for neutralization with two conformational dependent antibodies created using HPV18 VLPs—H18.K2 and H18.J4. The deletion mutant loses the ability to be neutralized with the H18.K2 antibody but is still neutralized by the conformation dependent H18.J4 antibody. The H18.K2 antibody was produced against VLPs, and therefore, the binding site cannot be contained in the 35 upstream amino acids. This suggests that the 35 upstream amino acids induce structural changes within the HPV18 capsid. When HPV16 virions produced in organotypic raft culture are run on an SDS-PAGE gel, two bands for L1 can be detected on the western blot [38,39,40,41]. In contrast, western blots of HPV16 QV appear to only have one form of L1 incorporated into virions, as only one band is visible [38]. This suggests both a potential conformational and structural difference between capsids produced in differentiated epithelium compared to those produced in monolayer cells. While the ability to quickly and easily produce infectious recombinant particles allows for quick experimentation, it is becoming clear that any important results generated should be verified utilizing native particles derived from differentiating epithelium.

## 3. Maturation and Assembly of Viral Particles

During the process of developing into fully infectious virions, many viruses undergo structural changes, called maturation [42,43]. In the raft culture system, virions mature over a period of 10–20 days within the tissue. In contrast, recombinant particles are matured over a period of 24 h after they are released from cells. After 10 days in foreskin epithelial raft culture, encapsidated and infectious virions are present, however, given another 10 days (20-day tissue) the virions are more mature as displayed by an increase in stability, defined by an enhanced resistance to stress imposed by fractionation in an ultracentrifuge [38]. This is similar to what has been seen for PsVs, with mature capsids having an increased stability [44]. Matured PsVs have also been shown to have an increased resistance to trypsin digestion and chemical reduction, as well as a more ordered structure when viewed by transmission electron microscopy (TEM) [44]. In addition to a more stable and mature phenotype, 20-day native virions were twice as infectious as 10-day virions and had an increased sensitivity to antibody neutralization by both L1 and L2 antibodies [38]. Both immature and mature PsV capsids were equally neutralized by many L1 and L2 antibodies [44]. This suggests that, for NV, the particles may be changing their exposure of key epitopes during the assembly and maturation process.

Analysis of both 10-day and 20-day tissue sections show a difference in virion localization with 10-day virions predominantly localized in the nuclei of suprabasal cells and 20-day virions being found mainly in the cornified layers of the epithelium. Human epithelium has a natural redox gradient with the lower part of the tissue being a reducing environment and the upper layers being an oxidizing environment [38]. This natural redox gradient coincides with the location of immature and mature virions, leading to the conclusion that virion maturation occurs when virions move from a reducing environment to an oxidizing environment. When an oxidizing agent, oxidized glutathione (GSSG) was added to the tissue during virion production, 10-day virions exhibited increased maturation [38,45]. This suggests that movement to an oxidizing environment induces changes in the viral capsid, converting it to a more stable and mature phenotype. PsVs are matured in an oxidizing environment in the presence of cellular lysate after being released from cells. Incubation of particles with cellular lysate, but not clarified lysate produced more mature particles, indicating that currently unknown cellular factors, the same of which are presumably present in tissue, are required for maturation as well [46].

While not absolutely essential for particle formation, a series of disulfide bonds in L1 and L2 molecules form both intra- and interpentameric interactions, stabilizing particles and making them more resistant to environmental influences from nucleases and proteases [44,47]. Within the epithelium, disulfide bond formation is thought to happen slowly during virion maturation in an oxidizing environment [38,44,47,48]. There are 12 conserved cysteine residues across L1 from multiple HPV types [48]. Evaluation of bovine papillomavirus (BPV) via cryoelectron microscopy showed that two separate disulfide bonds are present. However, only one of these is conserved among the human papillomaviruses [49]. Native and recombinant particles have both been reported to contain disulfide bonds. In HPV16, the disulfide bonding occurs at C175 and C428, forming an interpentameric bond between two neighboring capsomeres [50]. Based on mass spectrometry data for HPV18 VLPs, the homologous cysteines that participate in disulfide bonding are C175 and C429. Additionally, both genetic and biochemical analysis of HPV16 and HPV33 PsVs have shown the importance of these conserved cysteines in the structural integrity of the particles as mutation of the cysteines in HPV16 PsV prevents maturation of the particles [44,48]. Mutation and analysis of all 12 conserved cysteines in HPV16 VLPs identified three cysteines potentially involved in disulfide bonding—C175, C185, and C428. Mutation of C428 was especially detrimental to the capsid formation as only capsomeres were visible by EM. The C175S mutation produced tube like structures and the C185S mutation made smaller capsids [48]. These cysteine mutations were also evaluated in the context of NVs grown in differentiating epithelium [45]. Only the C175S mutation severely reduced production of infectious virions in both 10-day and 20-day tissue while C428S, C185S, and the C175S, C185S double mutation only affected 20-day virions. In contrast to the C428S PsV mutant, particle formation was observed for the C428S NV mutant. This suggests that in the context of differentiating epithelium, these cysteines are important for the formation of mature NV. Other conserved cysteines in HPV16 that do not play a role in interpentameric disulfide bonding were also evaluated. In HPV16 VLPs, cysteine to serine mutations of C161, C229, and C379, produced particles with a high susceptibility to tryptic proteolysis [48]. The same cysteine to serine substitutions in HPV16 NV hindered the accumulation of endonuclease resistant genomes in a stratifying epithelium with only the C229S mutant forming non-infectious virions. This suggests that these cysteines may be involved in forming transient disulfide bonds early in the assembly process to guide the capsid to its mature form [51]. Determining the precise roles of these cysteines in the HPV capsid may lead to be much better understanding of HPV capsid stability.

In addition to disulfide bonds in L1, L2 plays an important role in capsid formation and stabilization. L2 forms heterotypic interactions with L1 on the inside of the capsid as well as homotypic interactions with other L2 molecules. When L1 disulfide bonds were unable to form, L1 only capsomeres did not assemble into capsids. However when L2 was present, VLPs were formed [52]. Within the L2 terminus exists two highly conserved cysteines which, when mutated in PsVs, leads to production of non-infectious particles [53,54]. The importance of the L2 cysteines in particle formation was evaluated in the context of NVs produced in differentiating epithelium [55]. In contrast to previous studies using HPV16 PsV and QV, when one or both of the cysteines were mutated, infectious NVs were produced in both 10-day and 20-day tissue [53,54]. Evaluation of the mutant virions produced compared to wild-type showed that the mutants were more infectious but less stable. This could be due to enhanced presentation of a favored binding site on the capsid or a more effective release of viral genomes after host entry due to reduced capsid stability [53]. These L2 cysteines are in close proximity of the proposed external loop, which has been implicated as a feasible candidate for a cross neutralizing epitope across many HPV types for vaccine development. This loop is likely exposed in the final stages of virion maturation as only 20-day virions more efficiently neutralized with the L2 specific antibody RG-1 [38]. Other antibodies targeting this loop also only efficiently neutralized 20-day virions [38,39]. This data provides evidence for the importance of L2 in capsid stability and highlights a significant structural difference between virions assembled in monolayer *vs.* differentiating epithelium.

One of the remaining unknowns of HPV assembly and structure is the concentration of L2 in the viral capsid. L1 itself is able to self-assemble into capsids, however, including L2 into recombinant particles has been shown to increase yield, capsid stability, DNA encapsidation, and infectivity of particles [31,39,52,56,57,58]. Cryoelectron microscopy of native BPV1 suggests that there are 12 copies of L2 per capsid—one at the center of each of the pentameric capsomeres [59]. However SDS-PAGE analysis from native HPV1 particles, co-immunoprecipitation of HPV11 L1 pentamers with L2, as well as cryoelectron microscopy of HPV16 PsV suggests that up to 72 copies of L2 are present with one being at the center of each capsomere [59,60]. In the natural system, there is regulation as to the quantity of L1 and L2 produced. However, in an over expression system such as the ones used to produce recombinant particles, there would be no regulation of protein production and thus the ratio of L1 and L2 available for particle formation is likely skewed. This could allow for additional L2 to be incorporated into recombinant particles. Additional studies to identify the number of copies of L2 in native particles need to be done.

Organotypic raft culture allows for the generation of not only single base mutant virions but also for the generation of chimeric HPVs composed of different components of different HPV types in the natural environment of differentiating tissue. This whole gene replacement method has aimed at highlighting areas of conservation among different HPV types via complementation experiments. A chimeric virus, named HPV18/16, was created with the L1 and L2 ORF of HPV16 swapped into HPV18 in place of HPV18 L1 and L2 [61]. Substitution of both late gene ORFs did not affect viral genome maintenance in cells and raft cultures were found to have late gene functions such as capsid gene expression and virion morphogenesis. Additionally, chimeric virions purified from raft culture were able to infect keratinocytes [61]. As expected, the chimeric HPV18/16 was unable to be neutralized by HPV18 polyclonal antiserum but was neutralized by a HPV16 polyclonal antiserum. Additional chimeras created by swapping the L1s and L2s of more genetically diverse HPV types such as HPV45, 39, 31, 33, 11, 6b, 1a, CRPV, and BPV1 were also engineered. All the chimeras produced infectious virions, however, titers were much lower then those of wild-type HPV18 [62]. These data demonstrate that there are conserved biological functions between different HPV types. Similar studies illustrate that both capsid proteins play a role in the structure of each other and that this can ultimately affect the overall conformation and behavior of the virion [62,63]. Obviously, both L1 and L2 play an integral role in the biology of HPV.

In addition to L1 and L2, a variety of components comprise the viral capsid including viral DNA and histones. There are other possible viral and cellular components that either directly or indirectly affect the final structure and/or yield of capsids. These components could include HPV early proteins such as E2, E4, E5, and E7 or cellular proteins such as chaperones or karyopherins. There are undoubtedly unknown viral and/or cellular factors necessary for the assembly of native virions. These proteins could act by direction of proper subcellular localization, initial interaction of capsid proteins, mediation of the correct temporal formation of disulfide bonds, and regulation of capsid protein expression.

## 4. Attachment and Internalization

Our current understanding of the PV infection strategy proposes that, via microabrasions, virus particles gain access to the basal cells as well as the basement membrane. Utilizing recombinant particles, most papillomaviruses have been observed to infect cells by first binding to a glycosaminoglycan (GAG), heparan sulfate (HS), via L1 on either the basement membrane or the cell surface [64,65,66,67]. This binding event induces a conformational change allowing for the L2 N-terminus to be cleaved by a proprotein convertase (PC) such as furin or PC5/6 [68,69]. Additionally, these conformational changes may allow for interactions with secondary HS sites on the capsid, transfer the virion to an uptake receptor or receptor complex, and allow for exposure of hidden epitopes that may be important for the interaction with other cellular proteins in the entry process [69,70,71]. The involvement of GAGs as an attachment receptor was first demonstrated utilizing HPV11 L1 only VLPs and HaCaT cells. Not only was HPV shown to bind, but experiments suggested that L1 interactions with GAGs were GAG type specific [72]. Shortly thereafter, dependence on HS engagement was shown for both HPV16 and HPV33 PsVs [66]. However, in contrast to what was seen for HPV11 VLPs, both HPV16 and HPV33 PsV attachment was to a different subset of GAG molecules. This suggests either a difference between VLPs and PsVs or a difference between HPV types. While primary attachment to heparan sulfate proteoglycans (HSPGs) has been suggested to be a universal step for all PVs, noticeably, tissue derived HPV31 NV and HPV16 NV attachment to and infection of human keratinocytes was shown to be able to occur in the absence of HSPGs [15,73]. HPV31 NV infection of HaCaT cells, n-TERT-1 cells, and primary keratinocytes was unable to be blocked by exogenous heparin competition and could not be blocked by the enzymatic removal of cell surface HS. However, infection of Cos-7 cells and transformed C-33A cells was efficiently blocked by both methods [73]. These data indicate that the virus may use more than one receptor and/or entry pathway to enter a host cell depending on the cell type [73]. It is interesting to note that contradictory findings were observed for HPV31, where HPV31 NV was not dependent on HSPG for attachment, and HPV31 PsV was shown to be dependent on HS for attachment and infection of cells [67]. To complicate the story more, a recent report suggests that both HPV16 and HPV31 PsV attachment and infection can be independent of HSPGs if the non-HSPG molecule laminin-332 is present on the cell surface [14]. Possible explanations for these contradictions include the HPV type being studied, a possible structural variation between NV and PsV due to differences in maturation and assembly, and the cell types being utilized.

Due to minor structural differences between different virus types, it is possible that initial attachment to and interaction with the ECM and cell surface might be HPV type specific. Though the L1 protein displays overall sequence homology and structural conformation, both structural diversity and conformational differences in the loop structures of the pentamer surface have been demonstrated for HPV types 11, 16, 18 and 35 [74]. It is feasible that minor structural differences might have an impact on receptor engagement, including binding to attachment receptors as well as putative entry receptors. In a study looking at VLP binding for four different HPV types to the ECM and cell surface, some diversity was demonstrated [75]. Two more recent studies, one utilizing native virions [15] and one utilizing PsVs [14] also found HPV type dependent differences in ECM and cell surface binding. It is likely that slightly varying structures of different HPV types facilitate a preferred attachment to a specific GAG/GAG modification, laminin-332, or other unidentified receptors to initially bind to cells and trigger subsequent entry events. For the closely related polyomavirus, different types have been shown to utilize different receptors for infection [76,77,78]. Thus, a general hypothesis for attachment that encompasses all HPV types is not probable.

The entry process for HPV is slow, with cell surface events such as interaction with several receptors and conformational changes thought to be responsible [79,80]. HPV16 PsV and HPV18 PsV infection have been reported to have an average entry half time of 12 h in HaCaT cells [66,81]. Similarly, entry time for HPV31 NV as well as HPV31 QV was reported to be slow with a half time of about 14 h. In contrast, HPV16 QV was comparably rapid with an average entry half time of only 4 h in HaCaT cells [82]. This data suggests differences in HPV infections to be both HPV type and model system dependent. Several reports suggest that post engagement with cell surface HSPGs, HPV16 PsV particles require interaction with one or more non-HSPG receptors for internalization [69,81,83]. Post-attachment, there is initial co-localization of capsids with HSPGs on the cell surface. However, this co-localization is lost as the particles are internalized into intracellular vesicles, suggesting that particles have been transferred to a non-HSPG receptor [81]. Also giving strong evidence for the existence of other receptors is that furin pre-cleaved virions can bind to and infect HSPG negative cells [83]. Using biochemical inhibitors of endocytosis, it was suggested that HPV16 PsV and HPV58 PsV were internalized via clathrin-mediated endocytosis in COS-7 cells, while HPV31 PsV internalization was dependent on caveolae [84]. This is in contrast to a more recent study whereby HPV31 PsV was also reported to use clathrin mediated endocytosis in both COS-7 and 293TT cells [85]. When utilizing QV, caveolae mediated entry was demonstrated for HPV31 QV and HPV16 QV entry was again suggested to be mediated by clathrin coated pits [82]. HPV16 PsV infection has also been shown to be dependent on entry via caveolin-1 followed by particles trafficking to the ER upon entering cells [86,87]. In another system, this time using dominant negative inhibitors and siRNA knockdown in HeLa, 293TT, and HaCaT cells, the entry pathway of HPV16, HPV18, and HPV31 PsVs was described as a “clatharin, caveolin, lipid raft, flotillin, cholesterol, and dynamin independent mechanism distinct from macropinocytosis” [88,89]. The pathway was defined by a requirement for actin polymerization and tetraspanin microdomains with all particles trafficking to the late endosomal compartment with similar kinetics [89]. Taking into account the multiple HPV types studied, the virus system and cell type utilized, as well as the experimental approach taken, multiple pathways have been identified as playing a role in HPV entry. Considering the number of diverse HPV types, their virus tropisms, and the various HPV related diseases, it is possible that there is both overlapping and non-overlapping receptor usage that could feasibly shunt different HPV types into different entry pathways depending on cell type. It is also important to consider that when attachment assays are done utilizing recombinant particles, often a high multiplicity of infection (MOI) is used—typically in the thousands—which could be shunting particles into cells via pathways the virus would not typically use. In contrast, work with native virions typically uses MOIs of less than 100. Side-by-side comparisons should be done prior to making broad conclusions regarding the mechanism used for infectious entry. A more detailed analysis of the various HPV types using PsV, QV, and NV in relevant keratinocyte cell lines as well as in primary keratinocytes would both show similarities and highlight differences between the types that would help settle the growing amount of conflicting data in the literature.

To date, a multitude of receptors have been identified as potential primary and secondary HPV internalization receptors. One of the first identified was α6-integrin, an epithelial adhesion protein [90]. An α6-integrin specific monoclonal antibody was able to block HPV6b L1 only VLP binding to the cell surface by 60%. Additionally, HPV16 L1 only VLP binding correlated with α6-integrin in a study of 10 different cell lines [91]. However, cells deficient in α6-integrin can bind HPV16 L1 only VLPs and HPV11 NV infects α6-integrin negative cells at levels similar to an α6-integrin expressing cells [92]. In contrast to what is seen for VLPs, β4-integrin along with α6-integrin processing was found to be essential for infection of HaCaT cells with HPV16 PsV [93]. While it appears not to be absolutely essential for all HPV types, α6-integrin may contribute to infection efficiency, especially with its involvement in the Ras/MAPK and PI3K intracellular signaling pathways that are activated upon binding, which may function to create a more proliferative cellular environment to support infection [94]. A role for growth factor receptors (GFRs), both epidermal growth factor receptor (EGFR) and keratinocyte growth factor receptor (KGFR), has also been suggested [95]. Though not yet demonstrated, it is proposed that a HPV-HSPG complex binds directly to GFRs. While blocking EGFR inhibits infection of HaCaT cells with both HPV16 PsV, HPV31 PsV and HPV31 QV, the actual role for EGFR has yet to be determined [88,95]. It is possible that EGFR is directly involved in entry or it is possible that EGFR is activated and involved only in a signaling cascade. Another candidate, Annexin-A2 is a calcium and phospholipid binding protein that is expressed on the cell surface as a part of a heterotetramer, A2t, consisting of two Annexin-A2 monomers associated with a S100A10 dimer [96]. Both HPV16 VLPs and HPV16 PsVs directly interact with A2t, as shown by co-immunoprecipitation. Knockdown of Annexin-A2 by shRNA yields a significant decrease in internalization by HPV16 VLPs and infection by 16 PsV. Further, mutation of a site in L1 shown to be important for A2t binding reduces both binding and infection of HPV16 PsV [97]. HPV16 PsV is still able to infect Annexin-A2 deficient HepG2 cells, suggesting the use of an alternative infectious pathway [98]. While it will be important to discover the internalization receptor(s) for HPV, there is again a necessity for the consideration of HPV type, viral system, and cell line being utilized.

## 5. Subsequent Steps in HPV Infection

After endocytosis, acidification of the endosomal lumen allows for disassembly of the viral capsid and cellular sorting allows for transport to the nucleus where viral replication occurs [28,65,88,99,100]. Much of the L1 is dissociated from the L2-DNA complex with the help of cyclophillins [101]. Utilizing both HaCaT and HeLa cells, it has been shown that the L1-L2-DNA complex then travels to the Golgi via the retromer, a complex involved in transport of cellular cargo from the endosome to the Golgi [102,103,104,105,106,107]. This retrograde trafficking is required for HPV infection and HPV16 L2 has been shown to bind directly to the retromer to mediate escape from the endosome [103,108]. Utilizing BPV PsV, Syntaxin 18, which mediates trafficking of vesicles between the ER and the Golgi apparatus, has also been implicated in playing a role in trafficking the L2-DNA complex to the nucleus [109,110]. The L2-DNA complex then travels through the ER, requiring γ-secretase [87]. Inhibition of γ-secretase blocks infection of HPV after endosomal exit but prior to arrival in the Golgi [87]. Two separate HPV entry siRNA screens have identified ER components that are enriched during entry [102,103]. Additionally, both knockdown of specific ER proteins and use of chemical inhibitors of ER function inhibit HPV infection [102,111]. However, there are conflicting studies regarding ER co-localization with one study reporting co-localization of HPV with ER markers during infection [86] and the other failing to observe ER co-localization [104]. Finally, the L2-DNA complex trafficks to the nucleus, gaining entry during nuclear envelope breakdown during mitosis [27,102,112]. The L2 minor protein is essential for infection, as it facilitates transport of the HPV genome to the nucleus. Specifically, a transmembrane domain along with three GxxxG motifs within L2 have been shown to be essential for infectivity [113]. Much of the research looking at viral infection has been done utilizing PsV, which harbors a non-viral pseudogenome. While this allows for a much faster method of screening, results should be confirmed utilizing native virions.

In a natural infection, HPV infects keratinocytes. However, experiments *in vitro* rarely utilize primary keratinocytes. This is in part due to the extremely low infection efficiency of recombinant particles in primary cells [83]. Conversely, native virions infect primary cells quite well. HPV31 and HPV45 have been shown to infect primary cells just as efficiently as HaCaT cells and HPV16 infects primary cells with a greater efficiency compared to HaCaT cells [15]. HPV18 infects primary cells, however, with lower efficiency compared to HaCaT cell infection. This could be due to a difference in the expression of attachment or internalization receptors present on primary cells compared to other cells. Much of the data for HPV attachment and infection, whether using recombinant particles or native virions, is done in a variety of cell lines including, but not limited to, HaCaT cells, HeLa cells, 293TT cells, and CHO cells. The lack of utilizing a consistent cell line between experiments is likely responsible for some of the conflicting data when looking at both attachment and infectivity.

Of equal importance when looking at infection is the ability to look at virus neutralization. This is especially important due to the use of VLPs in the HPV vaccine that is now being administered to prevent infection. Almost all of the antibodies generated for HPV have been made using recombinant particles. Many of these antibodies generated, such as H16.V5 and H16.E70, have been shown to neutralize both recombinant particles and native virions [16,32,114,115]. However, some antibodies shown to be highly effective in neutralizing recombinant particles, such as H16.U4, have no neutralizing capabilities against native virions [32]. Also of significance are studies highlighting the mechanisms with which different antibodies neutralize particles. For example, H16.V5 and H16.E70 allow attachment of particles to the cell surface but blocks association with the ECM as well as internalization. H16.U4, however, prevented binding to the cell surface but not to the ECM [115]. These neutralization studies again highlight the probable structural differences between recombinant particles and native virions and the importance of confirming data using native virions.

## 6. Translational Research

There are two approved vaccines against the high-risk HPV types HPV16 and HPV18—Gardasil and Cervarix. Gardasil also provides protection against low-risk HPV types HPV6 and HPV11. These vaccines, however, are not efficiently cross protective against other high-risk HPV types. Therefore, another HPV vaccine, Gardasil 9 was recently approved that, in addition to the four types of HPV previously mentioned also provides protection against high-risk types HPV31, HPV33, HPV45, HP52, and HPV58. While these vaccines have been shown to be highly effective at preventing infection and the development of lesions, their high cost limits their use in developing countries. Additionally, the vaccine rate is low in some developed countries. Thus, the development of less expensive microbicides that would offer protection against a multitude of HPV types would offer women an additional protection against contracting HPV infection.

Due to the importance of GAGs, specifically HS, in HPV attachment to and infection of host cells, agents targeting GAGs are of great interest. Both high molecular weight sulfated or sulfonated polysaccharides and polymers such as cellulose sulfate, dextran sulfate, and polystyrene sulfonate showed microbicidal activity against BPV1 in mouse cells and HPV11 and HPV40 in human A431 cells without showing cellular toxicity [116]. More recently, Carrageenan, a highly sulfated polysaccharide derived from red algae, was identified in an *in vitro* screen of compounds that may effectively block infection by high-risk HPV types [117]. In this study, PsVs were used to determine whether there was a block in infection. However, when tested with NV, carrageenan failed to inhibit infection by HPV16 at concentrations up to 100 μg/mL. In contrast, significant levels of inhibition were observed at concentrations as low as 1 μg/mL for native HPV18. The IC_50_ for carrageenan inhibition of various HPV PsV types was in the ng/mL range [117]. In contrast, utilizing similar titers of NV, infection with HPV18 took at least 10 μg/mL of carrageenan for 50% inhibition and HPV16 was not inhibited. HPV31 NV was also found to be sensitive to inhibition by carrageenan and HPV45 showed no dose-dependent decrease in infection in the presence of carrageenan [15]. These results suggest that not only is there a difference in resistance and susceptibility of NV and PsV to the effects of carrageenan, but that different HPV types are very selective in their requirements for the type of GAG required for infection. Virion attachment to the cell surface in the presence of carrageenan was then analyzed. Attachment of HPV16 NV to the cell surface was unaffected in the presence of carrageenan. Conversely, HPV18 NV attachment was completely ablated in the presence of carrageenan. Additionally, HPV31 NV attachment was slightly reduced, whereas HPV45 attachment was not significantly reduced [15]. Taken together, these data suggest that when testing microbicides, confirmation of data utilizing native virions is essential. In addition, confirmation of results utilizing multiple HPV types should be done, as these data again confirm that not every HPV type behaves in the same manner.

Preventing the incidence of HPV infection through the use of vaccinations and microbicides is invaluable. Of equal importance is the use of chemical disinfectants to keep surfaces and medical equipment free of virions that could be spread between patients. Determining the efficacy of chemical disinfectants against HPV is important because it includes real world scenarios of disinfection protocols on equipment in hospital settings that are possibly contaminated with HPV. Currently, what little information that is available on HPV disinfection has been based mainly on studies from surrogate viruses, such as hepatitis B virus (HBV), which were deemed, without experimental evidence, to have similar resistance to HPV. Additionally, the United States Centers for Disease Control and Prevention (CDC) currently acknowledges the lack of research regarding effective HPV disinfectants [118]. Disinfectant information from studies that have included HPV has all been acquired utilizing recombinant HPV particles. Until recently, no studies had been done using native virions and there existed no functional assays to determine HPVs susceptibility to clinical disinfectants. A recent study tested the susceptibility of both HPV16 recombinant particles (QV) and native HPV16 grown in organotypic raft culture to a variety of common clinical disinfectants. Viral particles were incubated with common clinical disinfectants such as isopropanol, ethanol, triple phenolic, paracetic acid silver-based disinfectant (PAA), gluteraldehyde, hypochlorite, and ortho-phthalaldehyde. The disinfectant was then washed away and virus was incubated with cells to determine its ability to infect cells compared to untreated virus. HPV16 QV and HPV16 NV had some similarities in their resistance/susceptibility profiles to the disinfectants tested. Both types of particles were resistant to gluteraldehyde and ortho-phthalaldehyde and both were susceptible to hypochlorite and high concentration PAA. Of importance, however, HPV16 QV was also susceptible to isopropanol, triple phenolic, and a lower concentration of PAA [119]. This study highlights the necessity of utilizing native HPV particles when investigating clinical aspects of viral infection and transmission.

## 7. Concluding Remarks

Taken together, all of the data has provided great insight into the structure of HPV as well as the method of binding to, entering, and infecting cells. The use of recombinant particle technology is invaluable, as it allows for rapid data generation in studies looking at multiple parts of the HPV life cycle. However, as this review points out, there are some significant differences between native virions and recombinant particles that necessitate conformational studies utilizing native virions.
